# Levels of selected essential and non-essential metals in wheat (*Triticum aestivum*) flour in Ethiopia

**DOI:** 10.1017/jns.2022.70

**Published:** 2022-08-26

**Authors:** Wudineh Dessie Alemu, Alemu Lelago Bulta, Mesfin Bibiso Doda, Camerun Kastro Kanido

**Affiliations:** 1 Natural Disaster Risk Management Commission, Addis Ababa, Ethiopia; 2 Department of Chemistry, College of Natural & Computational Science, Wolita Sodo University, P.O. Box 138, Sodo, Ethiopia

**Keywords:** Cd, Cu, Fe, Flame atomic absorption spectrometer, Ni, Pb

## Abstract

In the present study, the levels and probable public health risks of selected metals (Fe, Mn, Cu, Zn, Ni, Cd and Pb) in nine wheat flour samples collected from Amhara, Oromia, South region, and the Strategic Food Reserve Agency were determined using FAAS and compared with results of prior studies and critical level. The wet digestion method using 65 % HNO_3_ and 72 % HClO_4_ in 300°C for 3 h was used when preparing the sample. Validation of the optimised digestion method was assessed using the spiking method, and an acceptable percent recovery from all metals. The levels of Fe, Cu, Mn, Zn, Ni and Cd ranged between 8⋅5297 and 11⋅1535, 1⋅633 and 4⋅2346, 3⋅1875 and 8⋅5313, 2⋅3589 and 2⋅7719, 0⋅154 and 0⋅854, and 0⋅0411 and 0⋅216 mg/kg, respectively, for Ethiopian wheat flour, while the level of Fe, Cu, Mn, Zn, Ni, Cd and Pb were ranged between 8⋅0099 and 8⋅1089, 1⋅663 and 1⋅6691, 4⋅5625 and 4⋅6250, 2⋅3015 and 2⋅3072, 0⋅9423 and 1⋅1346, 0⋅1593 and 0⋅1606, and 0⋅13 and 0⋅1381 mg/kg, respectively, for imported wheat flour. However, Pb had a concentration of less than 0⋅043 mg/kg for Ethiopian wheat flour. Findings indicate that Ethiopian wheat is comparatively higher in Fe, Mn, Cu, Zn and Cd, but lower in Ni and Pb than imports. From the result of the study, it can be concluded that the level of heavy metals determined in this study was within the permissible limit, and no probable health risk because both the Hazard quotient (HQ) and the Hazard Index (HI) are found to be below 1⋅0 regarding study metals.

## Introduction

Wheat (*Tritium aestivum*) is one of the most important grains in the world and is a staple food for over one-third of the world's population^(1)^. In Ethiopia, wheat is widely consumed and the most important cereal crop, particularly in urban areas. It provides about 15 % of the caloric intake for the countries with over 90 million populations, placing it second after maize and slightly ahead of teff, sorghum, and enset, which contribute 10–12 % each. After South Africa, Ethiopia is the second largest producer of wheat in Sub-Saharan Africa^(2)^.

Regular consumption of wheat and wheat flour products is essential for human beings to keep fit and continue all life processes. Wheat contains a wide variety of essential elements like sodium, potassium, iron, calcium, boron, magnesium, selenium, copper, zinc and others. The majorities of these metals are natural components of the earth's crust and present naturally in food or can enter food as a result of human activities such as industrial and agricultural processes. These components are essential in maintaining cellular processes. Other metallic elements do not have functional effects on the body and may be harmful to health if food containing them is often consumed in the diet^(3)^.

Over three billion people are currently micronutrient (i.e. micronutrient elements and vitamins) malnourished^(4)^. Trace essential metal deficiencies in humans exist in both developing and developed countries and may be considered ‘hidden hunger’^(5)^. This worldwide crisis in nutritional health is the result of dysfunctional food systems that do not consistently supply enough of these essential nutrients to meet the nutritional requirements of high-risk groups^(4)^. In developing countries, a large percentage of the population has no access to meet in their diet; the daily food intake is mostly cereal-based and does not support the microelement and vitamin needs of the population. These are the major cause of micronutrient malnutrition. Furthermore, their diet is often very monotonous, which increases the risk of inadequate dietary intake of one or more micronutrients^(5)^.

The essential micronutrients of wheat flour do not provide calories, but they play an important role in the metabolic regulations of the human body if they are present in required quantities. However, if the levels of these essential ingredients exceed acceptable limits in wheat and wheat products, they become detrimental to our health. The analysis of metals in food is a major aspect of food quality control^(6)^. They can play an important role in biochemical systems, constituting a significant threat to plant, animal and human system health^(6,7)^.

The trace elements (micronutrients) considered in this particular study are the minerals Mn, Fe, Ni, Cu and Zn. The daily allowances for these metals differ from one person to another according to the levels of development, gender as well as the norms of the different countries they set. According to the USA standards RDI of Mn, Fe, Cu and Zn are 2⋅3, 8, 0⋅9 and 11 mg/d for matured adults, and 2⋅6, 9, 1⋅3 and 13 mg/d for lactating females, respectively. However, the value of Fe exceeds the values indicated during pregnancy and becomes 27 mg/d^(8)^. The value of these elements was less than the FAO/WHO allowable limit as per^(9)^.

Metals such as mercury, lead, cadmium and arsenic are poisonous at very low levels^(10)^. The occurrence of toxic metals in industrial wastewater is of interest because they are often present at significant levels and if discharged into surface waters can have severe effects on the environment and public health. Thus, the presence of concentrations of non-essential metals in plant tissues leads to poisoning problems in humans and other animals that feed on specific plant tissues^(11)^. These toxic metals have a potentially harmful effect, not just on compounds, but also on human health. This is because of their cumulative properties and toxicity, although they are usually present in agricultural soils at low concentrations^(12)^. Heavy metal contamination in Ethiopia may be occurred due to irrigation with contaminated water, the addition of fertiliser and metal-based pesticides, industrial emission, transportation, harvesting process, storage and/or sale^(13)^.

A complete profile of essential and non-essential metals must be available for the nutritionist and consumers. A lot of work has been done on metal determination in foodstuff. However, data regarding mineral content is still lacking in developing countries like Ethiopia. Excessive levels could imply a risk as wheat products represent a high percentage of the Ethiopians’ diet^(9)^. Therefore, this study was initiated to determine the levels of essential and non-essential metals in wheat flour in Ethiopia. The results of the study serve as a springboard or benchmark for further research and further mineral analysis to conduct similar wheat flour research.

## Materials and methods

### Description of sampling locations

Wheat kernels were collected in various regions of Ethiopia and imported. The sampling sites for Ethiopian wheat were the major areas of wheat grain production, while different types were mainly consumed in the country and for comparison, the imported sample was used. The samples were collected from Western Gojjam, Arisi and Wolaita Zone, while imported samples were collected from Strategic Food Reserve Agency which had different sites, and were mixed as an imported sample. Samples other than imported and local were collected from the AARC and DZARC that were grown in 2017–18. Sampling sites and their locations are summarised in Table 1.

**Table 1.** The geographical location, sampling site characteristics and distance from Addis Ababa

District	Latitude (N)	Longitude (E)	Altitude (m)	Distance (km)	Reference
AARC	11°16′32″	37°29′30″	2240	567	(14)
DZARC	08°44′N	38°58′E	1900	47	(14)
Wolaita	6°51″ and 7°35″	37°46″ and 38°1″	1200	383	(15)

### Sample collection

1 kg of each of the four-wheat varieties (Tay, Ogolcho, Mossobo and Selam) which were collected from different parts of Western Gojjam (Amhara region) were taken from Adet Agricultural Research Center. Similarly, 1 kg of each of the three-wheat varieties (Kakaba, Obontu and Yerer) which were collected from different parts of the Arsi zone (Oromia region) were taken from Debre Zeite Agricultural Research Center. 1 kg of the wheat sample (local variety) was also taken from the Wolaita zone (SNNPRS). 1 kg of imported wheat was also sampled from the Strategic Food Reserve Agency's site in Wolaita. Within each wheat variety, three samples were collected using a judgmental sampling technique. All individual samples were reduced to 0⋅5 kg as a result of the coning and quartering (sample divider) process. Following this, 1 g of each wheat sample was used for analysis. Finally, all samples were labelled in a polyethylene bag based on the production site and brought to the laboratory for sample preparation.

### Instruments and apparatus

A drying oven (Digital Heat, J.P. Selecta, Spain) was used to dry-washed wheat grain samples. A stainless steel mixing machine (Moulindex, France) was used to grind and powder the dried wheat sample. A digital analytical balance (Mettler Toledo, Switzerland) with ±0⋅000 g precision was used to measure the wheat samples. 100 ml round bottom flask with reflux condensers were used in the Kjeldahl heater (Gallenkamp, England) to digest samples of dried wheat flour and powdered wheat flour. Measuring bottles (Duran, Germany), beakers, volumetric vials and pipettes were used to measure different volumes of sample solution, acid reagents and metal standard solutions. Buck Scientific Model 210VGP AAS, East Norway, USA flame atomic absorption spectrometer equipped with deuterium arc background correctors and hollow cathode lamps with air-acetylene flame was used for the determination of Mn, Fe, Ni, Cu, Zn, Pb and Cd in wheat flour sample.

### Chemicals and reagents

All reagents and standards used in the study were of analytical quality. 65 % HNO_3_ (Merch, BDH, Germany) and 72 % HClO_4_ (Aldrich, A.C.S. reagent, Germany) was used to digest wheat flour samples for the determination of metals. Stock Standard solutions containing 1000 mg/l in 2 % of HNO_3_ of the metals, Mn, Fe, Ni, Cu, Zn, Pb and Cd were used to prepare calibration standards. Distilled water was used for the dilution of wheat samples and the preparation of metallic working standard solutions before analysis and the rinsing of glassware.

### Sample preparation for metal analysis

The sample preparation procedure typically consists of homogenisation and cleaning. Wheat samples were cleaned and washed with distilled water to remove dust and other impurities. The washed samples were oven-dried at a temperature of 80°C for 24 h until constant weight. The dried samples were grounded in a stainless steel blender and the powder samples were allowed to pass through a 1⋅2 mm sieve to remove the large particles. Finally, wheat flour is stored in a dry, clean, well-packed plastic polyethylene bag before digestion is carried out and brought to the laboratory^(16)^.

### Digestion of wheat sample

1 g of the sieved wheat flour sample was exactly weighed and transferred to a 100 ml round bottom vial. To this, 5 ml of HNO_3_ (65 %) and 1 ml of HClO_4_ (72 %) were added to each sample and the mixture was digested on a Kjeldahl digestion apparatus (Gallenkamp, England) fitting the flask to a reflux condenser at 300°C for an optimised period of 3 h. The digested solutions were left to cool for 30 min without disassembling the condenser from the flask and for 10 min after the condenser was removed. In the cooled solution, 50 ml of distilled water was added and smoothly agitated to dissolve the precipitate. The cooled digested samples were filtered into a standard 100 ml volumetric flask using Whatman filter paper (70 mm) to extract the insoluble solid from the supernatant liquid. To this 100 ml volumetric flask, distilled water was filled up to mark. In this stage, the solution was clear and colourless. The digested and diluted sample solutions were then kept in a refrigerator (Hitachi, Japan) and ready for further analysis^(17)^. To determine the concentration of metals in the acid mixtures, blank samples were digested and carried out in the same way as the wheat flour samples^(18)^. Digested and diluted sample solutions were measured using FAAS^(19)^.

### Instrument calibration

The Buck Scientific Model 210VGP AAS (East Norway, USA) has been calibrated using five sets of working standards. The standard working solutions of each metal (Mn, Fe, Cu, Zn, Ni, Cd and Pb) were freshly prepared through a series of dilutions. An intermediate standard solution of 10 mg/l of each metal was prepared by dilution of a 1000 mg/l stock standard solution. The prepared solution was used to calibrate the instrument before determining the concentrations of metals in the samples^(20)^. The operating conditions of the instruments and the detection limit of the given method are presented in Table 2.

**Table 2.** Analytical parameters for the determination of metals in wheat flour samples by FAAS

Metal	Wavelength (nm)	MDL (mg/kg)	Correlation coefficient (*R*)	Equation for calibration curve
Fe	248⋅3	0⋅718	0⋅9986	*y* = 0⋅0404*x* – 0⋅0026
Cu	324⋅7	0⋅237	0⋅9982	*y* = 0⋅1641*x* – 0⋅1309
Mn	279⋅5	0⋅515	0⋅9977	*y* = 0⋅0320*x* – 0⋅0050
Zn	213⋅9	0⋅057	0⋅9989	*y* = 0⋅3486*x* – 0⋅0673
Ni	232⋅0	0⋅034	0⋅9951	*y* = 0⋅0104*x* – 0⋅0018
Cd	228⋅9	0⋅029	0⋅9975	*y* = 0⋅1588*x* – 0⋅0253
Pb	283⋅2	0⋅073	0⋅9966	*y* = 0⋅042*x* – 0⋅0006

MDL, method detection limit.

### Method detection limit

The method detection limit (MDL) is the smallest amount of a substance in a given matrix that can be measured with a 99 % confidence level that the analyte concentration is greater than zero. In the present study, six blank samples were used and the pooled standard deviation was calculated. The MDL was computed by multiplying the pooled standard deviation of the blank sample by three^(16)^. Method detection limits for the metals of interest, analytical wavelengths, correlation coefficients and correlation equations for metal calibration curves in wheat samples using FAAS are given in Table 2. The correlation coefficients for all the calibration plots were >0⋅9951 and they showed that there was a very good agreement between the concentration and the measured signal. The detection limits of the method are low enough to detect trace metals^(17)^.

### Method validation for metal determination

The spiked samples were prepared through the addition of a small known quantity of metal standard solutions. In the absence of certified reference material for the wheat flour samples, the validity of the optimised procedure was checked by adding the known concentration of each metal in 1 g sample from the stock solution (1000 mg/l) as follows: Standard solution of 1000 mg/l was used and 2⋅32 mg/kg of Mn, 5⋅15 mg/kg of Fe, 0⋅82 mg/kg of Cu, 0⋅75 mg/kg of Zn, 0⋅55 mg/kg of Ni, 0⋅025 mg/kg of Cd and 0⋅05 mg/kg of Pb were added into 100 ml volumetric flask that containing 1 g of wheat flour sample. The spiked and unspiked samples (blank during the recovery assay) were digested under similar conditions in triplicate^(17)^.

### Assessment of human health risk

Chronic daily intake (CDI) is the mass per unit of body weight (BW) of a substance per time averaged over a lifelong time. The human non-carcinogenic risk effects for each metal were assessed using equation (1) which shows the CDI (mg/(kg d)).1
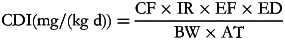


In this equation, CF is the average concentration of heavy metals in the sample (mg/kg), IR is the average daily intake rate of wheat product (kg/(person day)) which is 100 g/d in Ethiopia (www.ephi.gov.et), EF is the exposure frequency (365 days per year) and ED is the exposure duration (the mean life expectancy of a person is 67 years), BW is the average BW (70 kg taken for adults) and AT*_n_* is the mean exposure period for non-carcinogens (365 days per year × exposure number per year)^(21,22)^.

Hazard quotient (HQ) has been used to characterise the risk to human health by the intake of metal-contaminated food^(23)^. HQ indicates lifetime non-carcinogenic risk. HQ is the ratio between exposure and the reference oral dose (RfD). The RfD values (mg/(kg d)) used in the assessment were: Mn: 0⋅1, Fe: 0⋅7, Ni: 0⋅02, Cu: 0⋅04, Zn: 0⋅3, Cd: 0⋅001 provided by^(24)^. If CDI increases, the HQ increases, and if HQ exceeds 1⋅0, there is cause for concern. If the ratio is lower than one, then there is no apparent risk. An estimate of the potential hazard to human health (HQ) through consumption of wheat grain grown in metal-contaminated is described in the following equation^(22,25)^.2
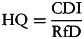


To evaluate the potential risk to human health through more than one heavy metal, the Hazard Index (HI) has been developed by Environmental Protection Agency for Health Risk Assessment of Chemical Mixtures^(22)^. The HI is the sum of the hazard quotients assuming that the magnitude of the adverse effect will be proportional to the sum of multiple metal exposures. It also assumes similar working mechanisms that linearly affect the target organ. When the HI exceeds 1⋅0, there should be a concern for potential health effects, and if it is below 1⋅0, it shows health benefits for consumption of wheat flour and that the consumers are safe^(26)^.3
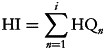


### Data analysis

The data analyses were carried out via the statistical analysis system. The wheat flour data generated were subjected to analysis of variance (ANOVA) using the general linear model procedure. The least significant difference (LSD) test was used to determine the differences among wheat flour samples from different sites based on the level of metals at *P* = 0⋅05. The simple correlation analysis of data was computed to determine the relationship between each metal level with other metal levels in the wheat flour.

## Results and discussion

### Recovery result

The percentage recovery of the metals studied in wheat flour ranged from 90 to 109 % (100 ± 10 %) for all metals (Table 3). Therefore, this verifies that the optimised digestion procedures and instruments were valid for metal determinations in wheat flour and are believed to remove metal fractions associated with organic matter^(27)^.

**Table 3.** Recovery test results for the optimised procedure in wheat flour (*n* = 9, mg/kg)

Metals	Concentration of unspiked sample	Amount added	Concentration in spike	Recovery (%)
Fe	9⋅3905 ± 0⋅94	5⋅15	14⋅3345 ± 0⋅93	96
Cu	2⋅1243 ± 0⋅982	0⋅82	2⋅9935 ± 0⋅01	106
Mn	4⋅8241 ± 1⋅689	2⋅32	7⋅0281 ± 0⋅12	95
Zn	2⋅5682 ± 0⋅181	0⋅75	3⋅2657 ± 0⋅03	93
Ni	0⋅5760 ± 0⋅277	0⋅55	1⋅1755 ± 0⋅07	109
Cd	0⋅1671 ± 0⋅045	0⋅025	0⋅1901 ± 0⋅012	92
Pb	0⋅033 ± 0⋅002	0⋅05	0⋅078 ± 0⋅001	90

### Levels of metals in wheat flour samples

**Fig. 1.**
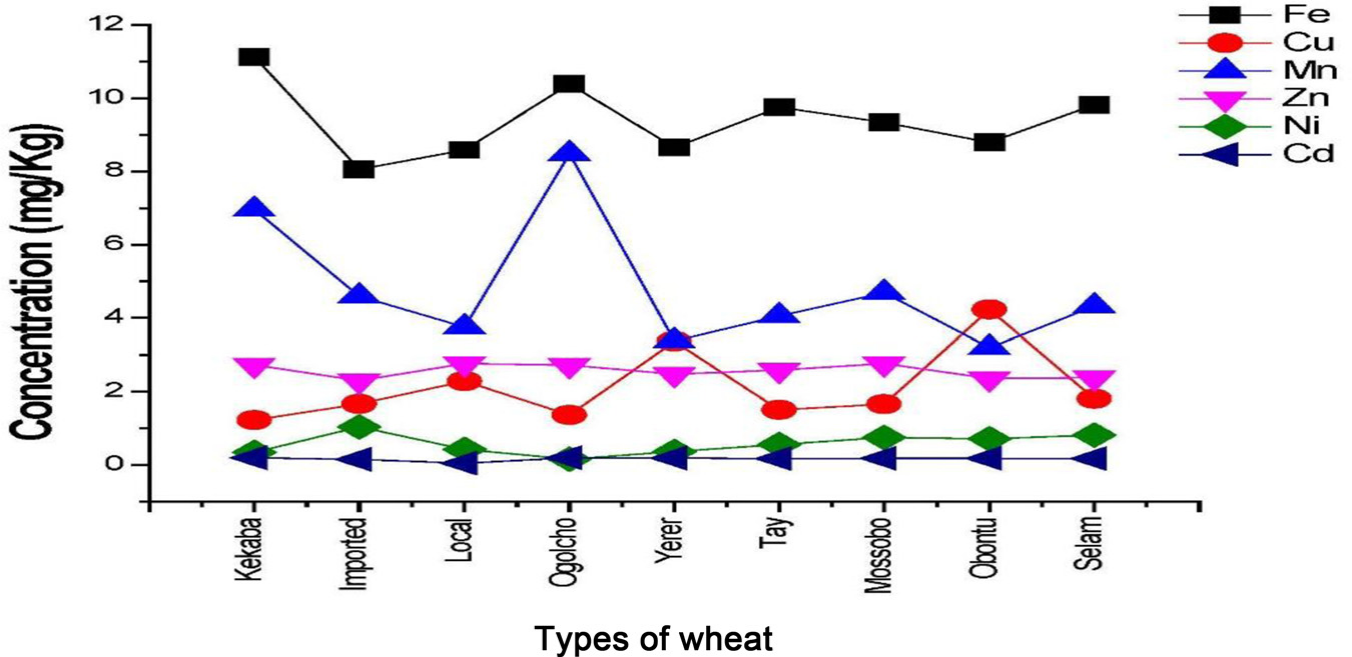
Distribution of metals in various types of wheat flour.

The wheat flour samples were analysed for essential metals (Fe, Cu, Mn, Zn and Ni) and non-essential metals (Cd and Pb) using FAAS. The mean values determined were triplicate analysis for each metal and the results were reported in terms of mean values ± sd for all metals in this study. Among all the determined metals, the concentration of Pb could not be expressed except for imported wheat because their amounts were below detection limits. The determined results and metals distribution pattern for each wheat flour variety are shown in Table 4 and Fig. 1.

**Table 4.** Mean concentrations (mean ± sd, *n* = 9 mg/kg) of essential and non-essential in wheat flour using one-way ANOVA at 95 % confidence level

Wheat variety	Metal concentration (mg/kg)						
	Fe	Cu	Mn	Zn	Ni	Cd	Pb
Kakaba	11⋅1287^a^ ± 0⋅025	1⋅2243^g^ ± 0⋅061	6⋅9584^b^ ± 0⋅018	2⋅7289^b^ ± 0⋅003	0⋅3425^e^ ± 0⋅038	0⋅1950^ba^ ± 0⋅026	ND
Imported	8⋅0594^g^ ± 0⋅050	1⋅6671^d^ ± 0⋅004	4⋅5938^d^ ± 0⋅031	2⋅30437^g^ ± 0⋅003	1⋅0385^a^ ± 0⋅096	0⋅1510^d^ ± 0⋅001	ND
Local	8⋅5875^f^ ± 0⋅052	2⋅2866^c^ ± 0⋅278	3⋅7500^g^ ± 0⋅0313	2⋅7662^a^ ± 0⋅006	0⋅4295^ed^ ± 0⋅055	0⋅0474^e^ ± 0⋅006	ND
Ogolcho	10⋅3861^b^ ± 0⋅05	1⋅3664^gf^ ± 0⋅0035	8⋅4792^a^ ± 0⋅048	2⋅7251^b^ ± 0⋅003	0⋅1637^f^ ± 0⋅009	0⋅1992^a^ ± 0⋅004	ND
Yerer	8⋅6535^f^ ± 0⋅0495	3⋅3713^b^ ± 0⋅009	3⋅3750^h^ ± 0⋅030	2⋅4822^d^ ± 0⋅003	0⋅3654^e^ ± 0⋅096	0⋅1866^bac^ ± 0⋅01	ND
Tay	9⋅7508^c^ ± 0⋅014	1⋅5026^ef^ ± 0⋅004	4⋅0521^f^ ± 0⋅036	2⋅5941^c^ ± 0⋅008	0⋅561^cd^ ± 0⋅2	0⋅1740^dc^ ± 0⋅004	ND
Mossobo	9⋅3383^d^ ± 0⋅038	1⋅661^ed^ ± 0⋅009	4⋅6875^c^ ± 0⋅0001	2⋅7672^a^ ± 0⋅006	0⋅7487^b^ ± 0⋅096	0⋅1849^bac^ ± 0⋅01	ND
Obontu	8⋅7937^e^ ± 0⋅038	4⋅2305^a^ ± 0⋅0035	3⋅1979^i^ ± 0⋅018	2⋅3627^f^ ± 0⋅004	0⋅7179^cb^ ± 0⋅056	0⋅1803^bc^ ± 0⋅004	ND
Selam	9⋅8168^c^ ± 0⋅043	1⋅8093^d^ ± 0⋅006	4⋅3229^e^ ± 0⋅018	2⋅3828^e^ ± 0⋅004	0⋅8167^b^ ± 0⋅058	0⋅17611^dc^ ± 0⋅001	ND

ND, not detected.

### Distribution pattern of metals

Metals uptake by plants depends on the ability to absorb metals from the soil, the accessibility of the mineral elements in both soluble and absorbable forms, the abundance of specific metals at the specified site and the contamination level of the soil with different types of metals. The variation of metals levels in soil arises because of increasing industrialisation and associated pollution of the biosphere, use of different types of agricultural fertilisers with different chemical compositions, pesticide treatment, use of sewage sludge, pesticides, geographic origin, irrigation of water and others has made some of the questionable quality for the production of food for humans and animals^(20,28)^.

#### Concentration of essential metals in wheat flour

Iron was the highest concentration followed by Mn among all samples except in Obontu, which contains a higher Cu concentration than Mn. The maximum concentration of Fe was found in Kakaba (11⋅1535 mg/kg) and the minimum in imported (8⋅0099 mg/kg) (Table 4). The average concentration of Fe obtained in this study was 9⋅3905 mg/kg, which is lower than the maximum permissible value (425 mg/kg) recommended by FAO/WHO^(9)^. In addition to this, the present study is also slightly lower than the literature value^(6)^. A higher level of Fe in this study among metals may probably result from the high concentration in the soil. Researches in recent years indicate that Ethiopian soil is high in Fe content, so wheat plants can absorb Fe easily^(27)^. This could be one reason behind the high value of Fe in Ethiopian wheat relative to other elements. Also, in acidic soil, the availability of Fe is high, and most of the wheat growing in Ethiopian soils is acidic^(29)^. The high amount of Fe in Ethiopian wheat flour in the present study may due to the acidic nature of the soil.

The present investigation revealed that the concentration of Cu varies from 1⋅1663 to 4⋅2346 mg/kg, which falls below the safe limits for human health. The highest concentration of Cu was found in Obontu (4⋅2346 mg/kg), while the lowest concentration (1⋅1663 mg/kg) was recorded in Kakaba. The mean concentration of the present study was recorded to be 2⋅1243 mg/kg. This value almost agrees with the reported value^(6)^ and is slightly higher than^(30)^. As shown in Table 4, the total accumulation of Cu in the order: Obontu > Yerer > Local > Selam > Imported > Mossobo > Tay > Ogolcho > Kakaba.

In the present investigation, the value of Mn ranges from 3⋅1875 to 8⋅5313 mg/kg among all samples. The maximum concentration (8⋅5313 mg/kg) of Mn was recorded in Ogolcho, while the minimum concentration (3⋅1875 mg/kg) was registered in the Obontu variety. In addition to this, the average concentration of Mn obtained in this study was (4⋅8241 mg/kg) which is much lower than the safe limit recommended by FAO/WHO^(9)^. Relatively higher Mn levels next to Fe among samples for the present study might be attributed to the availability of this micronutrient in acidic soils of the farmland. The availability of Mn present in the soil is known to depend on soil pH. The solubility of Mn becomes high at low pH due to this reason its concentration might be high in acidic soil^(28)^.

In the present study, the concentration of Zn was found to be high in Mossobo (2⋅7719 mg/kg), while a low concentration of Zn was observed in imported (2⋅3015 mg/kg). On the other hand, the average concentration was also 2⋅5682 mg/kg. This is lower than the safe limit recommended by FAO/WHO^(9,31)^. Zinc contents range from 2⋅3015 to 2⋅7719 mg/kg in the present study. However, these amounts were smaller than those reported by analyses of wheat flour made in Ethiopia, between 6⋅00 and 9⋅90 mg/kg; USA, between 7⋅0 and 7⋅2 mg/kg; Romania, 13⋅97 mg/kg^(32)^. The total accumulation of Zn in the order: Mossobo > Local > Kakaba > Ogolcho > Tay > Yerer > Selam > Obontu > Imported (Table 4).

In the present investigation, the maximum concentration of Ni (1⋅1346 mg/kg) was recorded in Imported, while the minimum concentration of Ni (0⋅154 mg/kg) was obtained in Ogolcho samples. The concentration of Ni ranges from 0⋅154 to 1⋅1346 mg/kg. On the other hand, the average concentration of the present study was 0⋅5760 mg/kg. However, this value was lower than the maximum permissible limit (67⋅9 mg/kg) recorded by FAO/WHO^(9)^. In the case of Ogolcho, the irrigation source may rain, which does not supply Ni. It could thus be supposed that Ni present in the soil came from municipal sewage or organic and synthetic manure applied to the soil. The low pH of the soil then facilitated plant uptake^(31)^. The concentration of nickel among wheat variety followed the trend: Imported > Selam > Mossobo > Obontu > Tay > Local > Yerer > Kakaba > Ogolcho. Most of the studied sites showed a more or less comparable pattern in mineral accumulation in the plant. Thus, the following pattern in decreasing order was noticed: Fe > Mn > Zn > Cu > Ni.

#### Concentration of non-essential metals in wheat flour

Cadmium is a non-essential element in foods and natural waters and accumulates predominantly in the kidneys and liver. Cadmium in food originates primarily from various sources of environmental contamination. Daily dietary exposure to Cd is estimated to be approximately 1⋅2 × 10^−4^ to 4⋅9 × 10^−4^ mg/kg BW. The dose of Cd in food should not exceed 0⋅007 mg/kg BW per week, according to the WHO^(20)^.

In the present investigation, the concentration of Cd was found to be high in Ogolcho (0⋅2124 mg/kg), while a low concentration of Cd was observed in local variety (0⋅0411 mg/kg). Cd levels vary from 0⋅0411 to 0⋅2124 mg/kg. On the other hand, the mean concentration for Cd is also 0⋅1671 mg/kg. This is below the FAO/WHO recommended threshold^(9)^. The Cd content of wheat or flour grains is influenced by many factors such as annual variation, cultivar differences, and genetic and regional disparities. Regional differences found in cadmium levels are mainly affected by cadmium deposition via the atmosphere, soil fertilisation and soil properties such as soil type, pH, and origin, and soil cadmium content^(31)^. The total accumulation of Cd in sequence: Ogolcho > Kakaba > Yerer > Mossobo > Obontu > Selam > Tay > Imported > Local (Table 4).

Lead is a major chemical contaminant in the environment and is very poisonous to humans. Pb may cause damage to the brain and kidneys, reduced haemoglobin production and male infertility. It enters the human body by inhalation and ingestion, absorbed and carried by the blood; it is accumulated in the liver, kidney and bone up to about the fifth decade of life. It also causes brain injury, particularly in young people. There is evidence that Pb pollution may induce aggressive behaviour in animals that may also occur in humans^(20)^.

In the present study, the concentration of Pb among the sampled varieties was below the detection limit, except for the imported sample (0⋅1357 mg/kg). This value was below the maximum allowable value declared by FAO/WHO^(9,31)^. In the previous report, the concentration of Pb in wheat was 0⋅107 mg/kg^(33)^ and 0⋅037 mg/kg^(30)^. The presence of Pb in imported wheat can be attributed to agriculture inputs containing Pb as an ingredient. On top of that, possibly because of the presence of factories that is tied to dyes in developed countries. Other causes of higher values may be relative exposure to contamination during storage and transport by cultivators^(16,34)^.

#### Comparison of metal levels in imported and Ethiopian wheat flour

From a broad perspective, most metals in imported wheat fall within the range of Ethiopian wheat (Table 5). The difference in the range of metals between imported wheat and Ethiopian wheat may suggest that the species varies. Since wheat imported from abroad belonged to the species bread wheat, Ethiopia wheat belonged to the species bread and durum wheat. Since imported wheat from abroad belonged to the species bread wheat, but wheat from Ethiopia belonged to the species both bread and durum wheat. In addition, the difference can also be attributed to variety, geographic location, soil pH, transportation, industrialisation and other factors^(17)^.

**Table 5.** Comparison of concentrations of metal (mean ± sd) for Ethiopian wheat with imported flour in mg/kg

Metal	Ethiopia wheat flour	Imported wheat flour	FAO/WHO (2001)
Fe	9⋅5569 ± 0⋅859	8⋅0594 ± 0⋅050	425⋅5
Cu	2⋅1815 ± 0⋅030	1⋅6671 ± 0⋅004	73⋅3
Mn	4⋅8529 ± 0⋅794	4⋅5938 ± 0⋅031	500
Zn	2⋅601 ± 0⋅164	2⋅3044 ± 0⋅003	99⋅4
Ni	0⋅5182 ± 0⋅234	1⋅0385 ± 0⋅096	67
Cd	0⋅1679 ± 0⋅048	0⋅1600 ± 0⋅0006	0⋅2
Pb	0⋅0203 ± 0⋅01	0⋅1357 ± 0⋅002	0⋅3

Mean concentrations of Fe, Cu, Mn, Zn and Cd for Ethiopian wheat flour are slightly higher than those of the imported sample, whereas mean concentrations of Ni and Pb are lower. Lead is not detected in all Ethiopian wheat flour varieties. However, the mean concentration of determined metals for both Ethiopian and imported samples was lower than the maximum permissible value recommended by FAO/WHO^(9,31)^ as shown in Table 5.

#### Comparison of metal levels of the present study with literature

Comparison of analytical data with reference materials is a common practice in analytical chemistry for validating findings. However, no standard reference material exists for comparing the results of these findings. We must therefore compare the results obtained with the investigations conducted in other countries by other investigators. The data presented in Table 6 indicated that most of the values reported in the literature were consistent with these studies. The level of iron in wheat flour used for comparison ranged from 0⋅02 to 13⋅61 mg/kg, while the value determined in the present study ranged from 8⋅0099 to 11⋅1535 mg/kg, which lies within the range. Similarly, the average concentration of Cu, Mn, Zn, Ni, Cd and Pb reported in the literature was in the order: 0⋅034–4⋅21, 4⋅309–7⋅66, 0⋅019–8⋅54, 0⋅006–0⋅27, 0⋅002–0⋅122 and ND–0⋅056 mg/kg, respectively^(6,9,33,35,36)^, while 1⋅1663–4⋅2346, 3⋅1875–8⋅5313, 2⋅3015–2⋅7719, 0⋅154–1⋅1346, 0⋅041–0⋅216 and ND–0⋅1381 mg/kg for Mn, Zn, Ni, Cd, and Pb, respectively, for the present study. Therefore, the levels were within the ranges indicated in the literature (Table 6).

**Table 6.** Comparison of the determined metal concentration (mg/kg, dry weight basis) in samples with reported values

Fe	Cu	Mn	Zn	Ni	Cd	Pb	Country	Reference
10⋅64	1⋅72	7⋅66	8⋅54	0⋅27	ND	0⋅05	Ethiopia	(9)
8⋅631 ± 1⋅717	2⋅275 ± 1⋅553	4⋅309 ± 0⋅187	6⋅154 ± 0⋅313	NR	0⋅027 ± 0⋅002	0⋅037 ± 0⋅013	Spain	(33)
8⋅168 ± 0⋅662	2⋅866 ± 0⋅981	4⋅414 ± 0⋅171	6⋅314 ± 0⋅211	NR	0⋅023 ± 0⋅002	0⋅056 ± 0⋅045	Spain	(33)
13⋅61	2⋅41	NR	11⋅32	NR	ND	NR	Nigeria	(6)
NR	4⋅21	NR	22⋅6	NR	0⋅122	0⋅024	Italy	(35)
0⋅04	0⋅016	ND	0⋅019	0⋅006	0⋅002	ND	Nigeria	(36)
0⋅02	0⋅034	ND	ND	0⋅06	0⋅004	ND	Nigeria	(36)
9⋅3905 ± 0⋅940	2⋅1243 ± 0⋅982	4⋅8241 ± 1⋅690	2⋅5682 ± 0⋅181	0⋅576 ± 0⋅277	0⋅1671 ± 0⋅045	ND	Ethiopia	Present study

ND, not detected; NR, not reported.

Generally, the observed metal concentrations were approximately comparable to the reported values. However, comparatively higher concentrations of Ni from essential metals and Cd from non-essential metals are observed in this study compared to reported values.

### Statistical analysis

One-way ANOVA was used to evaluate whether the difference in metal concentration between wheat samples was significant or not^(37)^. Except between Tay with Selam and Local with Yerer, there was a significant difference (*P* < 0⋅05) in mean concentrations of Fe at a 95 % confidence interval between all wheat flour varieties when the pairwise comparison was made. A significant difference (*P* < 0⋅05) was observed in average concentrations of Cu, except for Imported, Mossobo, Selam and also Ogolcho with Tay and Kakaba. However, there was a significant difference (*P* < 0⋅05) across all wheat flour varieties in the mean concentrations of Mn. The same was true for Zn except for Kakaba with Ogolcho and Local with Mossobo; there was a significant difference (*P* < 0⋅05) at a 95 % confidence interval among all wheat flour varieties. Similarly, significant variation was in Ni and Cd concentration for all the pairwise comparison tests except between Kakaba, Yerer, Local, and within Mossobo, Obontu, Selam for Ni, and between Ogolcho, Kakaba, Mossobo, Yerer, and within Selam, Tay and Obontu for Cd.

The absence of a significant difference in some mineral nutrients among wheat samples may indicate that these areas are under the same geographical location and share common climatic conditions^(37,38)^. Similarly, the presence of significant difference in concentration for some minerals of wheat flour might be due to the ability of the wheat plants to absorb metals from the soil, the availability of the mineral in the soluble forms, the abundance of specific metals at the particular site, the contamination level of soil with heavy metals, use of modern agricultural activities, i.e. the use of different fertilisers, pesticides, herbicides, varieties and species of wheat, the age of cultivation, soil pH, the use of sewage sludge, water irrigation and other. Therefore, the actual metal content of wheat probably varies for at least one of the aforementioned reasons^(31)^.

### Pearson correlation

In this particular study, to correlate the effect of the concentration of one metal on the other metal, the Pearson correlation coefficients (*r*) for the samples were used. The relationship between each metal in nine different types of wheat flour samples is shown in Table 7.

**Table 7.** Pearson's correlation for wheat flour samples

Metal	Fe	Cu	Mn	Zn	Ni	Cd	Pb
Fe	1						
Cu	−0⋅546	1					
Mn	0⋅733*	−0⋅648	1				
Zn	0⋅498*	−0⋅456	0⋅479*	1			
Ni	−0⋅534	0⋅079	−0⋅517	−0⋅660	1		
Cd	0⋅467*	−0⋅076	0⋅376*	−0⋅210	−0⋅025	1	
Pb	−0⋅512	−0⋅143	−0⋅116	−0⋅633	0⋅688*	−0⋅053	1

* Correlation is significant at the 0⋅05 level (two-tailed).

The values of Pearson correlation coefficient values in Table 7 showed a low and/or moderate positive correlation between metals, except for some metals. The weak correlation indicates that the presence or absence of one metal has less impact on the other metal. As we can see from Table 7, there is a relatively high negative correlation between Mn with Cu, Zn with Pb, and Zn with Ni in the majority of samples whereas Fe with Mn and Ni with Pb can be mentioned as strong positive correlations (Table 7). Fe with Zn, Mn with Cd, and Mn with Zn exhibit moderately positive correlations. Cu with Ni has low positive correlations while Zn with Cu, Mn with Pb and Zn with Cd have medium negative correlations. A weak negative correlation was also seen between Ni with Cd, Cu with Pb and Cd with Pb. These weak correlations may originate from common anthropogenic or natural sources as well as from differences in chemical properties^(39)^.

### The assessment of potential public health risks associated with wheat flour consumption

Potential health risk index values (CDI, HQ and HI) of different wheat flour samples were calculated for adults as presented in Table 8. The HQ values of all metals were below 1⋅0 which poses no public health risks. In addition, the HI values of wheat flour samples are lower than 1⋅0 and are thought to pose no probable public health risks (Table 8).

**Table 8.** Chronic daily intake (CDI), oral reference dose (RfD), hazard quotient (HQ) and total exposure hazard index (HI) for urban/rural wheat sample

Elements	Wheat type	CDI	RfD	HQ	HI
Fe	Kakaba	0⋅0030	0⋅7	0⋅00429	0⋅03187
	Imported	0⋅0021		0⋅0030	
	Local	0⋅0023		0⋅00329	
	Ogolcho	0⋅0027		0⋅00386	
	Yerer	0⋅0023		0⋅00329	
	Tay	0⋅0026		0⋅00371	
	Mossobo	0⋅0024		0⋅00343	
	Obontu	0⋅0023		0⋅00329	
	Selam	0⋅0026		0⋅00371	
Cu	Kakaba	0⋅00032	0⋅04	0⋅008	0⋅214
	Imported	0⋅00044		0⋅011	
	Local	0⋅00060		0⋅015	
	Ogolcho	0⋅00036		0⋅009	
	Yerer	0⋅00088		0⋅022	
	Tay	0⋅00039		0⋅0098	
	Mossobo	0⋅00044		0⋅011	
	Obontu	0⋅00111		0⋅028	
	Selam	0⋅00047		0⋅012	
Mn	Kakaba	0⋅00182	0⋅1	0⋅0182	0⋅1136
	Imported	0⋅00120		0⋅012	
	Local	0⋅00098		0⋅0098	
	Ogolcho	0⋅00222		0⋅0222	
	Yerer	0⋅00088		0⋅0088	
	Tay	0⋅00106		0⋅0106	
	Mossobo	0⋅00123		0⋅0123	
	Obontu	0⋅00084		0⋅0084	
	Selam	0⋅00113		0⋅0113	
Zn	Kakaba	0⋅00072	0⋅3	0⋅0024	0⋅05083
	Imported	0⋅00060		0⋅002	
	Local	0⋅00073		0⋅00243	
	Ogolcho	0⋅00071		0⋅0024	
	Yerer	0⋅00355		0⋅0118	
	Tay	0⋅00371		0⋅0124	
	Mossobo	0⋅00395		0⋅0132	
	Obontu	0⋅00062		0⋅0021	
	Selam	0⋅00063		0⋅0021	
Ni	Kakaba	0⋅000089	0⋅02	0⋅0045	0⋅09005
	Imported	0⋅000722		0⋅036	
	Local	0⋅000112		0⋅0055	
	Ogolcho	0⋅000043		0⋅002	
	Yerer	0⋅000095		0⋅0048	
	Tay	0⋅000147		0⋅00735	
	Mossobo	0⋅000196		0⋅0098	
	Obontu	0⋅000188		0⋅0094	
	Selam	0⋅000214		0⋅0107	
Cd	Kakaba	0⋅000051	0⋅001	0⋅051	0⋅391
	Imported	0⋅000040		0⋅040	
	Local	0⋅000012		0⋅012	
	Ogolcho	0⋅000052		0⋅052	
	Yerer	0⋅000049		0⋅049	
	Tay	0⋅000045		0⋅046	
	Mossobo	0⋅000048		0⋅048	
	Obontu	0⋅000047		0⋅047	
	Selam	0⋅000046		0⋅046	

## Conclusion

In the present study, the concentrations of metals (Fe, Mn, Zn, Cu, Ni, Cd and Pb) were determined in eight Ethiopian varieties and one imported wheat flour variety. The wet digestion method and the determination of selected trace metals in wheat flour using flame atomic absorption spectrometers have been shown to be effective in all minerals. The efficiency of sample preparation and digestion was tested in the recovery experiment. The levels of selected metals in wheat flour determined in this study can be expressed in the order: Fe > Mn > Zn > Cu > Ni > Cd > Pb (ND). The results of this work indicate that wheat flour accumulates relatively higher amounts of Fe and Cd among the determined essential and non-essential metals, respectively. Non-essential heavy metal, Pb, was detected only in samples of imported wheat, but not in samples of other wheat varieties. The contents of all minerals in wheat flour in this study were within the recommended limits and were free of toxic metals and therefore safe for daily human consumption. HQ and HI values were below 1⋅0 indicating that it would pose no public health problems. Thus, the study showed that the consumption of wheat flour is a good food source for treating different health complications.
